# Prolonging peripheral nerve block duration: Current techniques and future perspectives

**DOI:** 10.1111/aas.70010

**Published:** 2025-02-25

**Authors:** Mathias Maagaard, Eric Albrecht, Ole Mathiesen

**Affiliations:** ^1^ Centre for Anaesthesiological Research, Department of Anaesthesiology Zealand University Hospital Køge Denmark; ^2^ Department of Anaesthesiology and Intensive Care Copenhagen University Hospital – North‐Zealand Hillerød Denmark; ^3^ Department of Anaesthesiology University Hospital of Lausanne and University of Lausanne Lausanne Switzerland; ^4^ Department of Clinical Medicine University of Copenhagen Copenhagen Denmark

Peripheral nerve blocks are widely used for anaesthesia in upper and lower limb surgeries and as part of postoperative multimodal analgesia for various procedures. However, their primary limitation lies in their relatively short duration, which typically ranges from 8 to 14 h when using long‐acting local anaesthetics such as ropivacaine or bupivacaine.^1^ Achieving longer‐lasting effects has been a key area of research, leading to the exploration of various adjuncts,^1^, ^2^ liposomal bupivacaine^3^ and catheter‐based techniques.^4^

## 1 ADJUNCTS TO PROLONG NERVE BLOCKS

Numerous adjuncts have been tested to extend the duration of peripheral nerve blocks. These include epinephrine, magnesium, ketamine and midazolam. However, concerns over safety, inconsistent efficacy, or both have limited their adoption in clinical practice.^1^, ^2^ Buprenorphine, a partial opioid agonist, has been shown to prolong block duration by up to 9 h but is associated with a 5‐fold increased risk of postoperative nausea and vomiting, leading to its limited use as an adjunct.^5^ The most promising agents for prolonging block duration are dexamethasone and dexmedetomidine.^6^, ^7^

### 1.1 Dexamethasone

Dexamethasone has a well‐established safety profile, making it a widely used and reliable option. A single dose of dexamethasone perioperatively is not associated with increased risks of infection, delayed wound healing or important changes in blood glucose.^8^, ^9^, ^10^ Dexamethasone has been investigated as a perineural (injected with the local anaesthetic around the target nerve), intravenous and oral adjunct. Randomised trials and systematic reviews with meta‐analysis have consistently shown the efficacy of dexamethasone in prolonging peripheral nerve block duration by up to 7 h when compared with placebo.^7^

Research has been conflicting regarding whether the perineural or intravenous route is superior, but recent research indicated that the perineural and intravenous routes offer clinically similar block prolongation duration.^11^, ^12^, ^13^ A trial using oral dexamethasone (12–24 mg) with infraclavicular brachial plexus blocks demonstrated a 7‐h extension in block duration, comparable to intravenous administration.^14^ However, oral and intravenous routes of administration are yet to be compared directly in a clinical trial.

Perineural administration, although effective, is considered off‐label and presents concerns about compatibility with local anaesthetics due to the potential for crystallisation. Consequently, the intravenous route is preferred, with recommended doses ranging from 0.1 to 0.2 mg/kg.^1^

### 1.2 Dexmedetomidine

Dexmedetomidine, while also effective, is less favoured due to its associated adverse effects, including bradycardia, hypotension and sedation, which is not always advantageous in ambulatory surgery performed under regional anaesthesia only.^6^ Although one study reported a combination of dexmedetomidine and intravenous dexamethasone extending block duration to 66 h, subsequent trials failed to replicate this finding.^15^ Overall, dexamethasone, with its very safe and efficacious profile, is the preferred drug of choice for increasing block duration. Additionally, recent research has shown that dexamethasone is superior to dexmedetomidine by two hours.^16^

## 2 LIPOSOMAL BUPIVACAINE

Liposomal bupivacaine, approved by the FDA in 2011, represents a novel approach to prolonging block duration. It uses a liposomal formulation to facilitate the sustained release of bupivacaine. Liposomal bupivacaine represents the only real advancement in local anaesthetics since bupivacaine was first introduced in 1963 (albeit ropivacaine was introduced in 1996, it is very similar to bupivacaine). Despite early optimism and trials showing some benefits to plain bupivacaine or ropivacaine,^3^, ^17^ subsequent trials and systematic reviews have consistently not demonstrated its clinical superiority.^18^, ^19^, ^20^, ^21^ A notable concern is the high risk of for‐profit bias in the trials favouring liposomal bupivacaine, with nearly half of such studies showing positive results compared to just 11% of studies free from for‐profit bias.^22^ Additionally, a trial assessing the combination of liposomal and plain bupivacaine did not find clinically meaningful differences compared with plain bupivacaine alone.^23^

Furthermore, the addition of dexamethasone to plain bupivacaine seems to result in similar analgesic effects as liposomal bupivacaine,^24^ but at a fraction of the cost. For example, a single dose of 266 mg of liposomal bupivacaine is priced at approximately 334 USD, compared to 3 USD for plain bupivacaine.^22^

## 3 CATHETER‐BASED CONTINUOUS PERIPHERAL NERVE BLOCKS

Continuous peripheral nerve blocks facilitated by perineural catheters offer another strategy for prolongation of peripheral nerve blocks. These catheters enable continuous infusions or repeated boluses of local anaesthetic, theoretically allowing tailored anaesthesia for various procedures.^25^

While some studies have demonstrated superior analgesia with continuous nerve blocks compared to single‐shot techniques, particularly for shoulder surgery,^26^ others have shown limited benefits. For example, a recent randomised clinical trial assessing the addition of continuous versus single‐shot interscalene brachial plexus block to multimodal analgesia did not find the constant approach to significantly improve postoperative analgesia versus the single‐shot approach.^27^

Practical challenges also limit the widespread use of catheter‐based techniques. Catheter dislodgement is reported at rates from 5% to 25% for interscalene and femoral catheters, respectively.^28^ In a cohort of 1505 patients with an interscalene catheter at home, the incidence of catheter dislodgement was only 1.5%.^29^ Catheter‐based approaches are time‐consuming and require specialised expertise and support from an acute pain service for follow‐up. The risk of infection, estimated at around 3%, is another potential drawback.^30^

Despite these limitations, the catheter‐based approach remains valuable in specific cases where prolonged and adjustable analgesia is warranted. Further research into improving catheter designs and placement techniques could enhance their utility, particularly in an outpatient setting.

## 4 FUTURE PERSPECTIVES

A number of fundamental research questions regarding the prolonging of nerve blocks warrant further investigation. The mechanism of action by which dexamethasone influences block duration is not understood, although it is partly attributed to its anti‐inflammatory properties. Studies in animal models, both with and without surgical or traumatic inflammation, may provide valuable insights into the role of inflammation in block duration. Additionally, it is unclear what block duration or magnitude of increase in block duration is important to patients. While block efficacy has traditionally been evaluated based on duration‐related outcomes, incorporating quality of recovery scores may better capture patient‐important differences. Future research should focus on determining the minimal important differences for both block duration and quality of recovery in the context of regional anaesthesia. Comparative trials on the efficacy of oral versus intravenous dexamethasone are also warranted. Such studies would not only assess clinical outcomes but also allow for the assessment of the environmental impact of choosing oral over intravenous formulations.

The future for prolonging nerve block duration is promising and lies in several potential avenues: (1) testing additional or new drugs as potential adjuncts for increasing block duration; (2) development of next‐generation local anaesthetic formulations with longer duration of action; (3) improved catheter technologies with a novel design that reduces the risk of dislodgement, and a streamlined placement could increase the feasibility of home use, including removal without hospital contacts and (4) combination approaches, with trials investigating the synergistic effect of adjuncts, advanced formulations and catheter‐based techniques.

## 5 SUMMARY

Currently, intravenous or oral dexamethasone remains the adjunct of choice for prolonging peripheral nerve blocks based on efficacy, safety and cost‐effectiveness. Liposomal bupivacaine, while innovative, does not seem to provide any clinically important benefits over plain local anaesthetics, especially in the case of plain local anaesthetics with dexamethasone. Continuous peripheral nerve block techniques, while effective in some scenarios, face challenges related to time, expertise and follow‐up requirements.

Future advancements in local anaesthetics, adjunct therapies and catheter technology hold promise for overcoming the current limitations of single‐shot nerve blocks. Until then, it seems reasonable to proceed with the administration of long‐acting local anaesthetics with oral or intravenous dexamethasone for peripheral nerve blocks, along with the prescription of a multimodal analgesic regimen. The use of catheters depends on patient needs, available resources and clinical expertise.

## AUTHOR CONTRIBUTIONS

MM wrote the initial draft, and all authors participated in revising the manuscript.

## FUNDING INFORMATION

No funding was received for this manuscript.
